# Predicting Rotator Cuff Tears Using Data Mining and Bayesian Likelihood Ratios

**DOI:** 10.1371/journal.pone.0094917

**Published:** 2014-04-14

**Authors:** Hsueh-Yi Lu, Chen-Yuan Huang, Chwen-Tzeng Su, Chen-Chiang Lin

**Affiliations:** 1 Department of Industrial Engineering and Management, National Yunlin University of Science and Technology, Touliu, Yunlin, Taiwan; 2 Department of Orthopedics, National Taiwan University Hospital Yun-Lin Branch, Touliu, Yunlin, Taiwan; Mathematical Institute, Hungary

## Abstract

**Objectives:**

Rotator cuff tear is a common cause of shoulder diseases. Correct diagnosis of rotator cuff tears can save patients from further invasive, costly and painful tests. This study used predictive data mining and Bayesian theory to improve the accuracy of diagnosing rotator cuff tears by clinical examination alone.

**Methods:**

In this retrospective study, 169 patients who had a preliminary diagnosis of rotator cuff tear on the basis of clinical evaluation followed by confirmatory MRI between 2007 and 2011 were identified. MRI was used as a reference standard to classify rotator cuff tears. The predictor variable was the clinical assessment results, which consisted of 16 attributes. This study employed 2 data mining methods (ANN and the decision tree) and a statistical method (logistic regression) to classify the rotator cuff diagnosis into “tear” and “no tear” groups. Likelihood ratio and Bayesian theory were applied to estimate the probability of rotator cuff tears based on the results of the prediction models.

**Results:**

Our proposed data mining procedures outperformed the classic statistical method. The correction rate, sensitivity, specificity and area under the ROC curve of predicting a rotator cuff tear were statistical better in the ANN and decision tree models compared to logistic regression. Based on likelihood ratios derived from our prediction models, Fagan's nomogram could be constructed to assess the probability of a patient who has a rotator cuff tear using a pretest probability and a prediction result (tear or no tear).

**Conclusions:**

Our predictive data mining models, combined with likelihood ratios and Bayesian theory, appear to be good tools to classify rotator cuff tears as well as determine the probability of the presence of the disease to enhance diagnostic decision making for rotator cuff tears.

## Introduction

The rotator cuff consists of 4 muscles and their tendons that stabilize the shoulder joint. Rotator cuff injury, including tendon impingement or a tear, is a common source of shoulder pain, accounting for approximately 50% of major shoulder injuries [1], [2]. The incidence of tears may increase with age; especially in people aged 60 years and older [1], [3]. Patients who report shoulder pain and are diagnosed with a rotator cuff tear may require aggressive treatment or surgical intervention [4].

Currently, a rotator cuff tear is diagnosed using clinical examination and imaging tests. A preliminary diagnosis can be obtained by assessing the shoulder for tendon weakness and rotational ability [1]. Clinical examinations are noninvasive and inexpensive, and a diagnosis can be obtained immediately at the time of the appointment; however, the accuracy is dependent upon physician experience. If needed, an imaging test such as magnetic resonance imaging (MRI) can be performed to confirm or rule out the diagnosis. The gold standard for the diagnosis of a rotator cuff tear is a double contrast arthrogram, which has 86% sensitivity and 96% specificity, but it is an invasive, costly, and painful procedure [5].

Clinical physical findings are important to establish the diagnosis of a rotator cuff tear and determine the optimum treatment plan [4]. Many noninvasive examination techniques have been developed to aid in diagnosing specific rotator cuff conditions [1]. In drop-arm test, patients are asked to elevate their arm fully and then slowly reverse the motion. If the arm drops suddenly or the patient experiences severe pain, the test is considered positive. This test shows good specificity (75%∼95%), but low sensitivity (10%∼35%) [6], [7]. Jobe test [7], [8] can suggest supraspinatus tendon impingement as well as test shoulder strength. By elevating the arm in the scapular plane and positioning the arm in full internal rotation, the function of the supraspinatus muscle can be partially isolated. Previous research show that the sensitivity and specificity of Jobe test for detecting rotator cuff tears were ranged from 40%∼90% and 65%∼80, respectively.

According to a systematic review from Health Technology Assessment, no clear national guidelines exist for the diagnosis of shoulder pain, and there is no definitive evidence that any single test can conclusively diagnose rotator cuff disorders [9]. Longo et al reported that the combination or sequence of clinical tests for the examination of shoulder disorders remains unclear [6]. Because the treatment of shoulder pain is different when a rotator cuff tear is present, obtaining a clinical diagnosis is important to make cost-effective treatment decisions [4], [10]; however, making the diagnosis can be difficult. The severity of rotator cuff injuries diagnosed clinically may not correlate with the severity determined by imaging tests [11]. In addition, research shows that diagnoses made by clinical examination alone have high false-positive rates [12], indicating that a large proportion of shoulder injuries diagnosed as rotator cuff tears by clinical exam are found to be normal on imaging tests. This may be due to the difficulty of confidently ruling out the diagnosis by exam alone.

Data mining is the computational process of discovering patterns or classifications in large datasets using a combination of artificial intelligence, machine learning, statistics, and database systems [13], [14]. This knowledge discovery methodology has become a popular research tool in different fields and been increasingly used in medical literatures to identify and exploit relationships among medical variables and predict outcomes of diseases using historical medical data [14], [15]. For some diseases, determining the diagnosis, prognosis or treatment planning is a primary challenging task for doctors and thus the predictive data mining model is a useful tool to use patient-specific information to predict an outcome of interest at an individual patient level and support clinical decision-making [14], [16]. Predictive data mining methods, such as artificial neural networks (ANNs) and decision trees, have been used successfully to predict the outcomes of medical diagnostic processes [14], [17]; Examples include identification of patients at high risk of postinduction hypotension during general anesthesia [18], prediction of acute coronary occlusion, early diagnosis of acute myocardial infarction [19], [20], prediction of thalassemic pathologies [21], diagnosis of ovarian cancer [22], and prediction of outcomes following treatment of internal shoulder derangements [23].

It would be beneficial to develop a diagnostic approach for rotator cuff tears that integrates and interprets clinical information without overusing imaging tests. Imaging tests should be reserved for expanding the clinical hypothesis or further clinical finding such as the tear size rather than being used to gain certainty in the diagnosis [24]–[26]. This study used predictive data mining methodologies and Bayesian theory to improve the accuracy of diagnosing rotator cuff tears by clinical examination alone. We developed and compared 3 predictive models (ANN, logistic regression, and decision tree) used to classify rotator cuff tears based on patient demographics, symptom history, and physical examination results. The likelihood ratio (LR) and Bayesian theory were then used to estimate the probability of a rotator cuff tear based on the results of the predictive models. We anticipated this approach would improve the ability to correctly diagnose a rotator cuff tear without overusing invasive and expensive imaging tests.

## Methods

In this retrospective study, 169 patients who had a preliminary diagnosis of rotator cuff tear on the basis of clinical evaluation followed by confirmatory MRI between 2007 and 2011 at the Department of Orthopedics, National Taiwan University Hospital Yun-Lin Branch were identified. MRI was used as a reference standard to classify rotator cuff tears. This study was approved by the National Taiwan University Hospital's Institutional Review Board (IRB case #201206066RIC). Patient consent was specifically waived by the approving IRB because this was a retrospective study in which patient information was de-identified before analysis by the researchers.

The outcome variable, namely, the MRI imaging result, was coded into a binary system of “tear” and “no tear.” The “tear” category included both partial- and full-thickness tears, and “no tear” was classified as normal. Rather than including an intermediate “partial-thickness tear” group, dichotomous results were used to reduce the false-positive rate (shoulders diagnosed as having a rotator cuff tear but found to be normal on MRI). The distribution of the outcome variable was 132 and 37 patients for “tear” and “no tear,” respectively, giving a false-positive rate of 22%. This rate was close to that of previous studies, in which the false-positive rate ranged from 10% to 30% [7], [27]. Because the “no tear” patients were underrepresented, an over-sampling approach [28] was used to balance the dataset. The predictor variable was the clinical assessment results, which consisted of 16 attributes (Table 1). Because Jobe test and drop-arm test are very common provocative tests in diagnosing rotator cuff tear [6], [7], those two tests were used as predictor variables in the predictive models.

**Table 1:** Variable list.

Variables	Type	Coding
Outcome		
Rotator cuff tear	Nominal	2 codes (1 for tear, 0 for no tear)
predictor		
Age	Ordinal	3 codes (1 for ≤ 45, 2 for 46∼65, 3 for > = 66)
Gender	Nominal	2 codes (1 for male, 0 for female)
Pain Index	Ordinal	7 codes (0 no pain ∼ 6 severe)
Injury side	Nominal	2 codes (1 for right, 0 for left)
Able to wear clothes	Nominal	2 codes (1 for yes, 0 for no)
Injury history	Nominal	2 codes (1 for yes, 0 for no)
Night pain	Nominal	2 codes (1 for yes, 0 for no)
Taking medicine	Nominal	2 codes (1 for yes, 0 for no)
Drop arm test	Nominal	2 codes (1 for positive, 0 for negative)
Jobe test	Nominal	2 codes (1 for positive, 0 for negative)
Range of motion test	Nominal	2 codes (1 for positive, 0 for negative)
Sharp pain	Nominal	2 codes (1 for yes, 0 for no)
Aching pain	Nominal	2 codes (1 for yes, 0 for no)
Throbbing pain	Nominal	2 codes (1 for yes, 0 for no)
Numbing pain	Nominal	2 codes (1 for yes, 0 for no)
Distending pain	Nominal	2 codes (1 for yes, 0 for no)

This study employed 2 data mining methods (ANN and the decision tree) and a statistical method (logistic regression) to classify the rotator cuff diagnosis into “tear” and “no tear” groups.

### ANN

The ANN was developed using the structure of multi-layer perceptron (MLP) with back-propagation (a supervised learning algorithm). It is a mathematical construct that uses previously solved examples to build a system of neurons to make new decisions, classify and forecast [29]. Because of its good predictive performance, ANN is a popular artificial intelligence-based data-mining algorithm used in clinical medicine [30]. Clinical diagnosis was one of the first areas in which ANN was applied [31].

### Decision Tree

A decision tree is a tree-like graph used to display decisions and their possible outcomes. It consists of nodes linked to 2 or more sub-trees and leaves [32]. The nodes of a decision tree represent predictor variables with each node having a number of branches equal to the number of values. The leaves represent the decision classes. A decision tree can provide highly accurate classifications presented as a simple representation of the data, making interpretation and determination of rules very easy [33]. Its effectiveness in many well-developed classification algorithms such as ID3, C4.5, C5 [32], [34], and CART [35] has resulted in its widespread use in medical research [33]. In this study, we chose to use the C4.5 algorithm as our decision tree method.

### Logistic Regression

Logistic regression is a generalized linear regression model widely used to predict the occurrence of an event [29]. It is used with increasing frequency in the health sciences because of its ability to model dichotomous outcomes. Logistic regression analysis was used in this study to obtain the coefficients for risk variables included in the logistic model [36].

**Figure 1:**
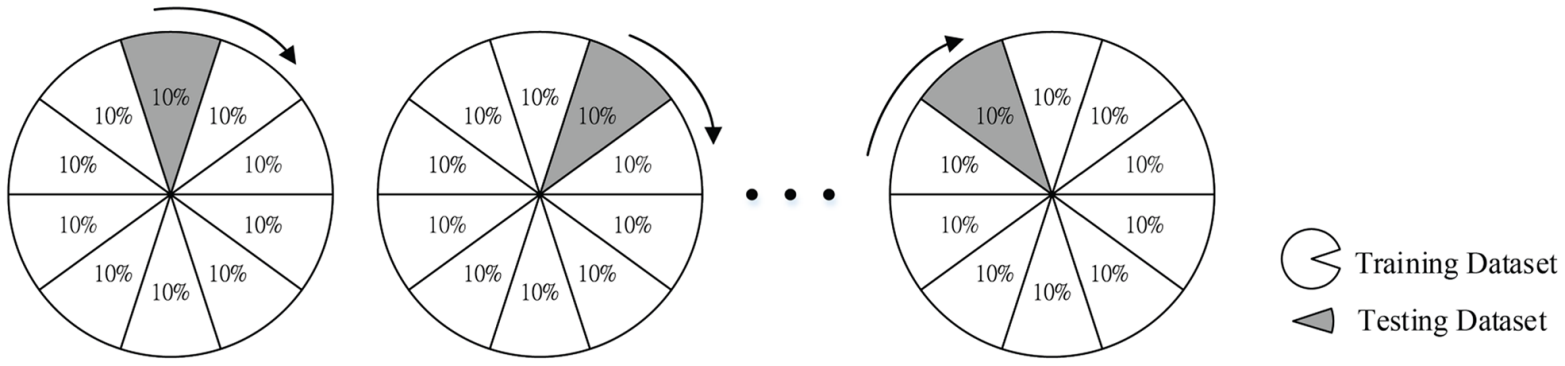
10-fold cross-validation.

To minimize the generalization error associated with randomness that leads to a biased estimation of future examples, the *k*-fold cross-validation is often used to validate the ability of a prediction model to generalize unseen data [37], [38]. *K*-fold cross-validation is a computational technique that randomly divides all sampling data into *k* equal size subsamples. One subsample is used as the validation for testing the model, and the remaining subsets are used as training data. The training and testing process is then repeated *k* times, with each subsample used as the validation data once. The subsample results are averaged, giving a single estimated error rate for unseen data. This estimate assumes that the original dataset is a random sampling of the population. It shows the ability to lower the prediction variance and avoid the bias of over-fitting on the training data [37], [39], [40]. In our study, 10-fold cross-validation was selected because many studies have shown that 10 is an optimal folding number considering the efficiency of completing the models [37], [41]. In the 10-fold cross-validation, the entire dataset was partitioned into 10 nonoverlapping subsets, and the procedure was repeated 10 times with different training and testing datasets (Figure. 1).

We used 3 performance measures (correction rate, sensitivity, and specificity) in comparative analysis to test the generalized error associated with the different predictive models. In addition, the area under the receiver operating characteristic curve (AUROC) was adopted as a measure to analyze discrimination power, which refers to the ability to distinguish those who have a tear from those who do not.

## Results

This retrospective study collected 169 subjects who were diagnosed with rotator cuff tears after clinical examinations. The demographic data were summarized in Table 2. The majority of subjects were female (57.4%); the ranges in age were between 16 and 82 years (mean age, 58.8 years; SD, 11.6 years); most injury side was on the right rotator cuff (65.7%) and nearly 60 percent of subjects rated their pain as level 4 (pain ranged from 1∼10). More than half of patients had injury history (54.4%), ability to wear clothes (66.9%) and night pain problem (84.6%). Two types of clinical examinations, Jobe test and drop arm test were considered as predictor variables to determine the rotator cuff tear, the percentage of positive diagnosis were 80.5% and 48.5% respectively (Table 3). The predictor variables also included five types of pain symptoms (sharp, aching, throbbing, numbing, and distending pain) which were coded in yes/no dichotomous responses (Table 3). Most patients mentioned having sharp pains (85.2%) and throbbing pains (72.2%). Some patients had aching pains (46.2%), numbing pains (5.9%) and distending pains (3.6%).

**Table 2:** Demographic variables of the 169 patients who were diagnosed with suspected rotator cull tear by the clinical examinations.

Description	Characteristics	Frequency	Percentage
Gender	Male	72	42.6%
	Female	97	57.4%
Patient age (M 58.8; SD 11.60)	16–45	17	10.1%
	46–65	105	62.1%
	66–82	47	27.8%
Injury side	Left	58	34.3%
	Right	111	65.7%
Pain index	2	2	1.2%
	3	47	27.8%
	4	100	59.2%
	5	18	10.7%
	6	2	1.2%
Injury history	Yes	92	54.4%
	No	77	45.6%
Able to wear clothes	Yes	113	66.9%
	No	56	33.1%
Night pain	Yes	143	84.6%
	No	26	15.4%
Taking medicine	Yes	165	97.6%
	No	4	2.4%
Rotator cuff tear (MRI test)	Yes	132	78.1%
	No	37	21.9%

**Table 3:** Symptoms related variables of the 169 patients who were diagnosed with suspected rotator cull tear by the clinical examinations.

Variables	Type	Frequency	Percentage
Drop arm test	Positive	136	80.5%
	Negative	33	19.5%
Jobe test	Positive	109	64.5%
	Negative	60	35.5%
Range of motion test	Positive	82	48.5%
	Negative	87	51.5%
Sharp pain	Yes	144	85.2%
	No	25	14.8%
Aching pain	Yes	78	46.2%
	No	91	53.8%
Throbbing pain	Yes	122	72.2%
	No	47	27.8%
Numbing pain	Yes	10	5.9%
	No	159	94.1%
Distending pain	Yes	6	3.6%
	No	163	96.4%

The P values were assessed to examine the similarity between the tear and no tear groups of each predictor variables (Table 4). Able to wear (*p* = 0.038) and Jobe test (*p* = 0.001) showed statistically significant difference between two groups. Others had no statistical significance.

**Table 4:** Characteristics of the 169 patients categorized as tear and no tear groups using MRI imaging as a reference standard.

Variable	No tear (n = 37)	Tear (n = 132)	P value
Age (years)	59.30 (17.211)	58.62 (9.640)	0.755
Gender (male/female)	15/22	57/75	0.774
Injury side (left/right)	17/20	41/91	0.092
Ability to wear (yes/no)	30/7	83/49	0.038
Injury history (yes/no)	18/19	74/58	0.424
Pain index	3.86 (0.673)	3.82 (0.675)	0.710
Night pain (yes/no)	30/7	113/19	0.500
Drop arm test (+/−)	29/8	107/25	0.716
Jobe test (+/−)	15/22	94/38	0.001
Range of motion test (+/−)	19/18	63/69	0.697
Taking medicine (yes/no)	35/2	130/2	0.169
Sharp pain (yes/no)	9/28	16/116	0.065
Aching pain (yes/no)	15/22	63/69	0.438
Throbbing pain (yes/no)	29/8	93/39	0.342
Numbing pain (yes/no)	3/37	7/125	0.523
Distending pain (yes/no)	0/37	6/126	0.187

T-test for continuous variable and Pearson Chi-square test for dichotomous variable.

Two data mining techniques (ANN and decision tree C4.5) and one statistics method (logistic regression) were employed to classify the outcomes (tear/no tear). For each prediction model, 20 experiments with 10-fold cross-validation approach were conducted to minimize the bias associated with random sampling of training and test datasets as well as estimate the prediction performances [42]. Table 5 shows the prediction performances of correction rate, area under the ROC curve (AUC), sensitivity, and specificity. ANN model had most favorable correction rate (90%), AUC (94%), sensitivity (87%), specificity (95%), positive likelihood ratio (17.40), negative likelihood ratio (0.14) and diagnostic odds ratio (127.15). Decision tree also showed similar abilities to identify rotator cuff tear with sensitivity (83%) specificity (95%), likelihood ratios (13.50 for positive, 0.20 for negative). The predictive data mining models (decision tree, ANN) had statistically better performances (Table 5) than the statistical technique (logistic regression). The positive likelihood ratio (LR+), negative likelihood ratio (LR-), and diagnostic odds ratio (DOR) are summarized in the Table 6 to indicate the prediction power for each model (see Appendix S1).

**Table 5:** Prediction performance.

Model	Correction Rate	AUC	Sensitivity	Specificity
Logistic regression	0.71^a^ (0.09)^b^	0.77 (0.10)	0.72 (0.12)	0.71 (0.15)
Decision tree C4.5	0.88* (0.06)	0.90* (0.07)	0.83* (0.10)	0.95* (0.08)
ANN	0.90* (0.07)	0.94* (0.07)	0.87* (0.10)	0.95* (0.08)

a average of 20 repetitive 10-fold experiments.

b standard deviation.

* statistically significant (p<0.05) difference comparing to logistic regression model.

**Table 6:** Likelihood ratio.

Model	LR+	LR− a	DOR b
Logistic regression	2.29	0.42	5.45
Decision tree C4.5	13.50	0.20	66.79
ANN	17.40	0.14	127.15

a LR+, LR−: likelihood ratios for positive and negative results, respectively.

b Diagnostic odds ratio: a measure of the effectiveness of a diagnostic test.

## Discussion

In this study, our proposed data mining procedures outperformed the classic statistical method. The correction rate, sensitivity, and specificity of predicting a rotator cuff tear were statistical better in the ANN and decision tree models compared to logistic regression. The results were analogous to previous studies that showed data mining techniques are potentially more effective than conventional statistical methods for analyzing the ability to accurately diagnose various diseases [43], [44]. However, predictive data mining has rarely been used by orthopedic surgeons for diagnosis. This limited acceptance may be due, in part, to the lack of studies on data mining use in the orthopedic literature. Several studies have evaluated the ability of the physical exam to correctly diagnose rotator cuff tears. A wide ranges of sensitivities (40% to 98%) and specificities (50% to 98%) have been reported in studies evaluating the accuracy of physical examination in diagnosing rotator cuff tears [4], [6], [7]. We found that our data mining models (ANN and the decision tree) were accurate for detecting rotator cuff tears with a sensitivity of 83–87% and a specificity of 95%, which compared favorably with rates reported in previous studies. Its moderate sensitivity and high specificity favor the use of data mining over classic statistical methods when diagnosing a rotator cuff tear, to avoid unnecessary imaging tests by reducing the false-positive rate.

An important feature of our predictive data-mining model is the transfer of evidence-based clinical research from the general population to the individual patient. Traditionally, statistics analyze a group of individuals to reveal significant relationships among the variables in the population studied, at the expense of predicting outcomes on an individual level [44], [45]. During clinic appointments, doctors are pressured to synthesize complex clinical assessment variables, such as physical and lab examinations, to make diagnosis and treatment decisions. Traditional medical statistics, which were designed mainly to explore group data, generally cannot be applied when determining the medical diagnosis of a single individual. The search for a method of predicting a specific diagnosis based on an individual patient's characteristics is the trend in evidence-based statistics [44], [46]. Therefore, the predictive data mining models in our study are timely and useful for answering specific classification questions at the level of the individual patient.

During clinical evaluation, a frequently encountered problem is how to determine the probability of a disease based on the clinical information. However, the classification output of predictive data mining is generally expressed as dichotomous categorical values in which the individual subject is classified into one class without a degree of confidence that the patient is in the correct group. To overcome this limitation, we combined prediction results with LRs and Fagan's monogram to assess the probability of having a disease. An LR, which is how much more or less likely a patient with the disease is to have a specified result than a patient without the disease, is a convenient and increasingly used measure to report test or prediction results [47]–[49]. It is calculated based on the sensitivity and specificity of the prediction results (see Appendix S1) and represents the likelihood, or odds, that disease is present based on the results of a test [50], [51]. As showed in Table 5, the sensitivity and specificity of the ANN model were 87% and 95%, respectively, which gives a positive LR (LR+) of 17.40 and a negative LR (LR−) of 0.14 (Table 6). This mean that a patient with a rotator cuff tear is approximately 17.4 times more likely to have a positive test or examination result than one who does not. Conversely, a patient without a rotator cuff tear is approximately 7.1 times more likely to have a negative predict test or examination result than one with a rotator cuff tear. Prior studies suggest that an LR+ greater than 10 significantly increases the probability of a positive test when the disease is present, and an LR− less than 1 indicates a negative test is unlikely to occur in a patient with the disease [52].

Based on LRs derived from our prediction models, Bayes' theorem could be used to assess the probability of a patient who has a rotator cuff tear using a pretest probability and a prediction result (tear or no tear). When examining the rotator cuff, a clinician may begin with a rough estimate of the likelihood a patient has a rotator cuff tear, referred to as the pretest probability, based on the patient's symptoms and history and the prevalence of the disease before ordering an imaging test [53]. In Bayes' theorem, the LR is used to modify the pretest probability of having the disease after a test result is known [50], [54]. Once a patient is classified into the “tear” or “no tear” group, the pretest probability could be altered to the posttest probability, which is what clinicians are most interested in.

**Figure 2:**
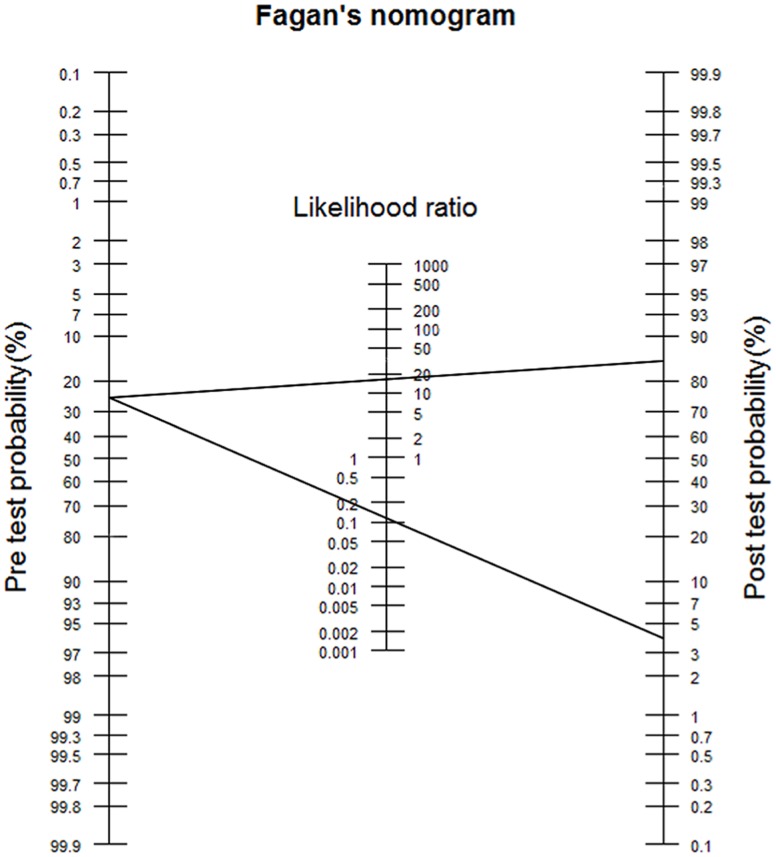
The use of the Fagan's nomogram (a straight line through the pretest probability of 25% and the LR+ of 17.40 yields a posttest probability of 85%; a straight line through the pretest probability of 25% and the LR- of 0.14 yields a posttest probability of 4%).

The posttest probability could be estimated using Fagan's nomogram, which is a graphical tool that easily estimates the posttest probability that a specific disease is present based on the result of a test and pretest probability [52]. As shown in Figure 2, a straight line starting with a pretest probability of having a rotator cuff tear, extended to the right of the LR, and intersecting with the posttest probability of having a tear. For example, if the prevalence rate of a rotator cuff tear for a patient is 25%, and our ANN model showed that this patient should be classified as a “tear” with an LR+ estimated at 17.40 (Table 6), a straight line (Figure 2) drawn from the pretest probability of 25% through the LR+ of 17.40 intersects with the posttest probability of approximately 85% (for calculations, see Appendix S1) [55]. This means that the probability of having a rotator cuff tear for this patient increases from 25% to 85% when the data mining result is “tear.” Alternately, when the data mining result is “no tear,” the probability of this patient having a tear decrease from 25% to 4%. Therefore, the results of our predictive data mining models could provide information to assist doctors in making diagnostic decisions, especially if the pretest probability of a rotator cuff tear is intermediate. Our predictive data mining results can be used not only to classify a patient into the “tear” or “no tear” category but also to modify the pretest probability in order to estimate the posttest probability, which is more useful information for making diagnostic and treatment decisions.

Further research is necessary to overcome the limitations of our study. First, to overcome generalization issue due to the sample size or variables selected [56], studies including additional patient characteristics or a larger study population are required. Second, more promising data mining methods such as support vector machines or Bayesian networks could be adopted to explore improvement of the prediction sensitivity and specificity. Third, further study is required to investigate whether other clinical evaluations such as the Hawkins test or the Neer test could be potential variables influencing the prediction performances. Although the actual pathology can only be determined by operative findings, our study did not use arthroscopy or open surgery as reference standards because it would have been unethical to perform surgery on all patients with a susceptive rotator cuff tear. Instead, we used less invasive tests (MRI) as a reference standard on all subjects. To reduce model verification bias, the validity and performance of our prediction models should be further evaluated using intraoperative findings as the gold standard for patients undergoing surgery.

## Conclusion

Currently the majority of orthopedists make a preliminary diagnosis of a rotator cuff tear based on physical examination; however, these examinations have a high false-positive rate, which leads to unnecessary imaging tests [12]. In this study, we developed 2 data mining models (ANN and a decision tree) and compared them using a statistical method (logistic regression) to determine the ability to predict the diagnosis of a rotator cuff tear based on 16 features of a physical examination. The classification results demonstrated that, when used to establish a preliminary diagnosis of a rotator cuff tear, the data mining models were superior to classic statistical methods on various performance indicators such as correction rate, sensitivity, and specificity. To our knowledge, this study is the first to retrospectively compare clinical examination alone with multiple personal characteristics (such as age, gender) and symptom history (such as pain index), which potentially influence the diagnosis of rotator cuff tears. In conclusion, our predictive data mining models, combined with an LR and Bayesian theory, appear to be good tools to classify rotator cuff tears as well as determine the probability of the presence of the disease to enhance diagnostic decision making for rotator cuff tears.

## Supporting Information

Appendix S1 ****
(DOCX)
