# Supplementary material for: Predicting Rotator Cuff Tears Using Data Mining and Bayesian Likelihood Ratios
Source: PLoS One. 2014 Apr 14;9(4):e94917. doi: 10.1371/journal.pone.0094917 (PMC3986413; doi:10.1371/journal.pone.0094917)
Supplement: Appendix S1 — (DOCX) [file pone.0094917.s001.docx]

# Appendix S1

Calculation of LR and DOR of Logistic regression model

(1)LR for a positive test result = sensitivity/(1–specificity)

(2)LR for a negative test result = (1–sensitivity)/specificity

(3)DOR = (sensitivity*specificity) / [(1-sensitivity)*(1-specificity)]

Conversion of pretest probability to pretest odds

Pretest odds = probability/(1-probability)

In example, pretest probability is 25%, thus the pretest odds is 25/(1-25)=0.33

Conversion of pretest odds to posttest odds after the test

Posttest odds= pretest odds*LR

In example, if the test is positive, the posttest odds=0.33*33.9=11.3

Conversion of posttest odds to posttest probability

Posttest probability = pretest odds/(1+ pretest odds)

In example, for a positive result, the posttest probability =11.3/(1+11.3)=0.92=92%.
